# The protective effect of neonatal oral administration of oleanolic acid against the subsequent development of fructose-induced metabolic dysfunction in male and female rats

**DOI:** 10.1186/s12986-018-0314-7

**Published:** 2018-11-20

**Authors:** Trevor T. Nyakudya, Emmanuel Mukwevho, Kennedy H. Erlwanger

**Affiliations:** 1 School of Physiology, Faculty of Health Sciences, University of the Witwatersrand, 7 York Road, Parktown, Johannesburg, 2193 South Africa; 2 Department of Biochemistry, Faculty of Natural Sciences & Agriculture, North West University, Mafikeng, Mmabatho, 2735 South Africa; 3 Department of Human Anatomy and Physiology, Faculty of Health Sciences, University of Johannesburg, Doornfontein, Johannesburg, 2028 South Africa

## Abstract

**Background:**

Consumption of fructose-rich diets has been implicated in the increasing global prevalence of metabolic syndrome (MetS). Interventions during periods of early ontogenic developmental plasticity can cause epigenetic changes which program metabolism for positive or negative health benefits later in life. The phytochemical oleanolic acid (OA) possesses anti-diabetic and anti-obesity effects. We investigated the potential protective effects of neonatal administration of OA on the subsequent development of high fructose diet-induced metabolic dysfunction in rats.

**Method:**

Male and female (*N* = 112) suckling rats were randomly assigned to four groups and administered orally: distilled water (DW), oleanolic acid (OA; 60 mg/kg), high-fructose solution (HF; 20% *w*/*v*) or OA + HF for 7 days. The rats were weaned onto normal commercial rat chow up to day 55. From day 56, half of the rats in each treatment group were continued on plain water and the rest on a high fructose solution as drinking fluid for 8 weeks. On day 110, the rats were subjected to an oral glucose tolerance test and then euthanased on day 112. Tissue and blood samples were collected to determine the effects of the treatments on visceral fat pad mass, fasting plasma levels of cholesterol, insulin, glucose, triglycerides, insulin resistance (HOMA-IR) and glucose tolerance.

**Results:**

Rats which consumed fructose as neonates and then later as adults (HF + F) and those which consumed fructose only in adulthood (DW + F) had significant increases in terminal body mass (females only), visceral fat mass (males and females), serum triglycerides (females only), epididymal fat (males only), fasting plasma glucose (males and females), impaired glucose metabolism (females only), β-cell dysfunction and insulin resistance (males and females) compared to the other treatment groups (*P* < 0.05). There were no differences in fasting serum cholesterol levels across all treatment groups in both male and female rats (*P* > 0.05).

**Conclusion:**

We conclude that neonatal oral administration of OA during the critical window of developmental plasticity protected against the development of health outcomes associated with fructose-induced metabolic disorders in the rats.

## Introduction

Metabolic syndrome (MetS), obesity and type 2 diabetes (T2DM) have increased to epidemic proportions worldwide in the last few decades [1]. Metabolic syndrome (MetS) is defined as a cluster of physiologic and metabolic disorders that are associated with a marked increase in the risk to develop T2DM and major adverse cardiovascular outcomes such as hypertension, atherosclerosis and myocardial infarction [2–4]. Obesity, on the other hand, is defined by the World Health Organization (WHO) as an increase in the mass of visceral adipose tissue which may or may not adversely affect health [5]. In fact, obesity is regarded as the main causative factor in the development of MetS and associated disorders such as T2DM [4].

Previously considered a problem in developed Western countries, the explosive increase in the global prevalence of metabolic disorders such as obesity, impaired glucose metabolism and dyslipidaemia now also pose a major burden and challenge to the public health sector in developing countries [6]. Worldwide, there are over 1.5 billion obese adults (over 20 years old) and 43 million children (under 5-years old) who are overweight [7]. Presently, it is estimated that over 415 million people are affected by T2DM worldwide, and according to the International Diabetes Federation, this figure is projected to rise to over 642 million by 2040 [8].

Genetic factors, age, ethnicity, urbanisation and lifestyle choices such as poor dietary habits and a decrease in physical activity have been implicated as the major contributing factors to the rise in the prevalence of health outcomes associated with metabolic disorders [9–11]. The increase in the consumption of high-energy diets, especially those containing fructose as the main ingredient, has also been linked to the increased incidence of obesity and metabolic dysfunction [12, 13]. A major source of commercial fructose is high fructose corn syrup (HFCS). HFCS is sweet and affordable and as such it is used as the main sweetener in several processed foods such as bread, yoghurts, cookies, breakfast cereals and sugar-sweetened beverages [10, 14].

Recent human epidemiological and rodent experimental studies have shown the developmental origins of metabolic disorders and associated diseases by establishing a link between the peri-conceptual, foetal or early infant phases of life and the subsequent development of adult obesity and the MetS [15–17]. The neonatal period has been identified as a critical window of developmental plasticity during which the nutritional status of the offspring can affect their subsequent development and confer epigenetic predisposition of the offspring to positive health outcomes or metabolic disorders later in life [18–21]. A growing number of clinical and experimental studies have shown that nutritional manipulations or stressful events during the neonatal period can influence epigenetic regulation of gene expression by changing the timing and direction of DNA methylation, histone modification and transcription of non-coding ribonucleic acid (ncRNA) [22–24]. The epigenetic phenomenon by which nutritional, hormonal, pharmacological and other stressful events acting in the critical periods of development, such as gestation and the neonatal period modify the development of certain physiological functions is known as neonatal programming [25, 26].

The vulnerability to the development of health outcomes associated with metabolic disorders such as T2DM and obesity is determined by exposure to adverse early life nutritional environments during gestation and the early postnatal period [16]. Maternal nutritional status during pregnancy and that of the offspring in the early neonatal period are also important in the development of metabolic disorders in the offspring [27]. The diet-induced epigenetic changes triggered in the neonatal period usually only become apparent later in life as either positive health outcomes or manifestations of metabolic disorders following a second hit or challenge [28]. Fructose consumption by the mother and/or offspring during the perinatal period has been associated with the development of metabolic disorders of the offspring later on in adulthood [29–31]. The metabolic developmental plasticity associated with the neonatal period makes it an important window of opportunity that can be targeted by pharmacological or dietary manipulations for prophylactic treatments that may result in the programming for positive health outcomes for the rest of the individual’s life.

In view of the increasing global prevalence of diet-induced metabolic disorders and the possibility of targeting the neonatal period for prophylactic treatments, alternative therapeutic interventions that can be administered in the neonatal period are needed to ameliorate the impact of the adverse health effects of obesity and MetS [32]. Most strategies used in the management of MetS emphasise on the use of pharmacological agents to manage or reduce the risk of developing health outcomes associated with obesity and metabolic dysfunction by targeting energy expenditure in adulthood [33]. However few studies have focussed on the neonatal period as a target for epigenetic modifications for the prevention of these metabolic conditions. Moreover prolonged pharmacological treatment of obesity and MetS may have adverse side-effects and reduces the quality of life for patients [34, 35]. The lack of patient compliance is also identified as having a negative outcome on the use of chronic medication [36].

Medicinal plants and herbs are used to prevent, treat and manage a wide range of diseases including metabolic disorders and their associated complications [33]. The therapeutic properties of medicinal plants can be attributed to the presence of phytochemicals which may act individually, additively or synergistically to improve health [37]. Oleanolic acid (3β-hydroxyolean-12-en-28-oic acid) is a biologically active pentacyclic triterpenoid phytochemical commonly found in several plant species that belong to the *Oleaceae* family such as olives, (*Olea europaea*) [38, 39]. Oleanolic acid (OA) is also found in virgin olive oil and fruits (apples and dates) [40, 41] and some commonly used medicinal plants (*Crataegus pinnafitida* and *Eclipta alba*) [42, 43]. Oleanolic acid possesses several potential pharmacological activities that exhibit several therapeutic activities without the adverse side effects of the commonly used synthetic pharmacological agents [44]. Oleanolic acid was used for the present study due to these previously described pharmacological activities which include hepatoprotection against chemical or fructose-induced liver injury [45, 46], anti-inflammatory properties [47, 48] anti-diabetic action [35, 44, 49, 50], anti-oxidant activities [51–54] and anti-glycosilative effects [55, 56]. Studies in adult animals have shown that OA possesses beneficial effects against the development of diabetes and MetS by preserving β-cell functionality and improving insulin sensitivity [44, 57, 58]. In rodent studies, OA has been demonstrated to improve lipid metabolism by downregulating he expression of lipogenic genes [59, 60]. OA also attenuates fructose-induced hyperglycaemia by modulating enzymes involved in carbohydrate digestion, insulin secretion and insulin signalling [58, 61].

In as much as OA has been shown to possess several therapeutic properties in the management of diet-induced metabolic disorders in adulthood, its potential protective effect against the development of health outcomes associated with fructose-induced metabolic syndrome later in life when administered in the neonatal period, a critical window of developmental plasticity needs to be investigated. Rats are altricial species consequently, the neonatal period provides a window for ontogenic plasticity and represents a viable interventional phase for neonatal programming.

## Methods

The study, conducted in the Central Animal Services (CAS) unit at the Faculty of Health Sciences, University of the Witwatersrand, was done in accordance with the International Standards of Care and Use of Animals in Research and was approved by the Animal Ethics Screening Committee (AESC) of the University of the Witwatersrand, Johannesburg, South Africa (Ethics clearance number: 2014/47/D).

### Housing and animal husbandry

Sprague Dawley (*Rattus norvegicus*) dams, each with between 8 and 12 pups, were used in this study. The rats were supplied by the CAS, University of the Witwatersrand, South Africa. Each dam and its respective litter were housed in acrylic cages in which wood shavings were used as bedding. The bedding was changed once a week. The ambient environmental room temperature was maintained at 25 ± 2 °C and a 12-h light and dark cycle followed (with lights on at 07:00 am). Adequate ventilation of the room was maintained at all times. The dams were supplied with standard commercially sourced rat chow (SRC) (Epol®, Johannesburg, South Africa) and water ad libitum throughout the suckling period. The dams were allowed to freely nurse until weaning of the pups on postnatal day (PD) 21. The dams were returned to stock after weaning of their offspring rats. The weaned rats were housed individually as described for the dams above.

### Study design and dietary treatments

**Fig. 1:**
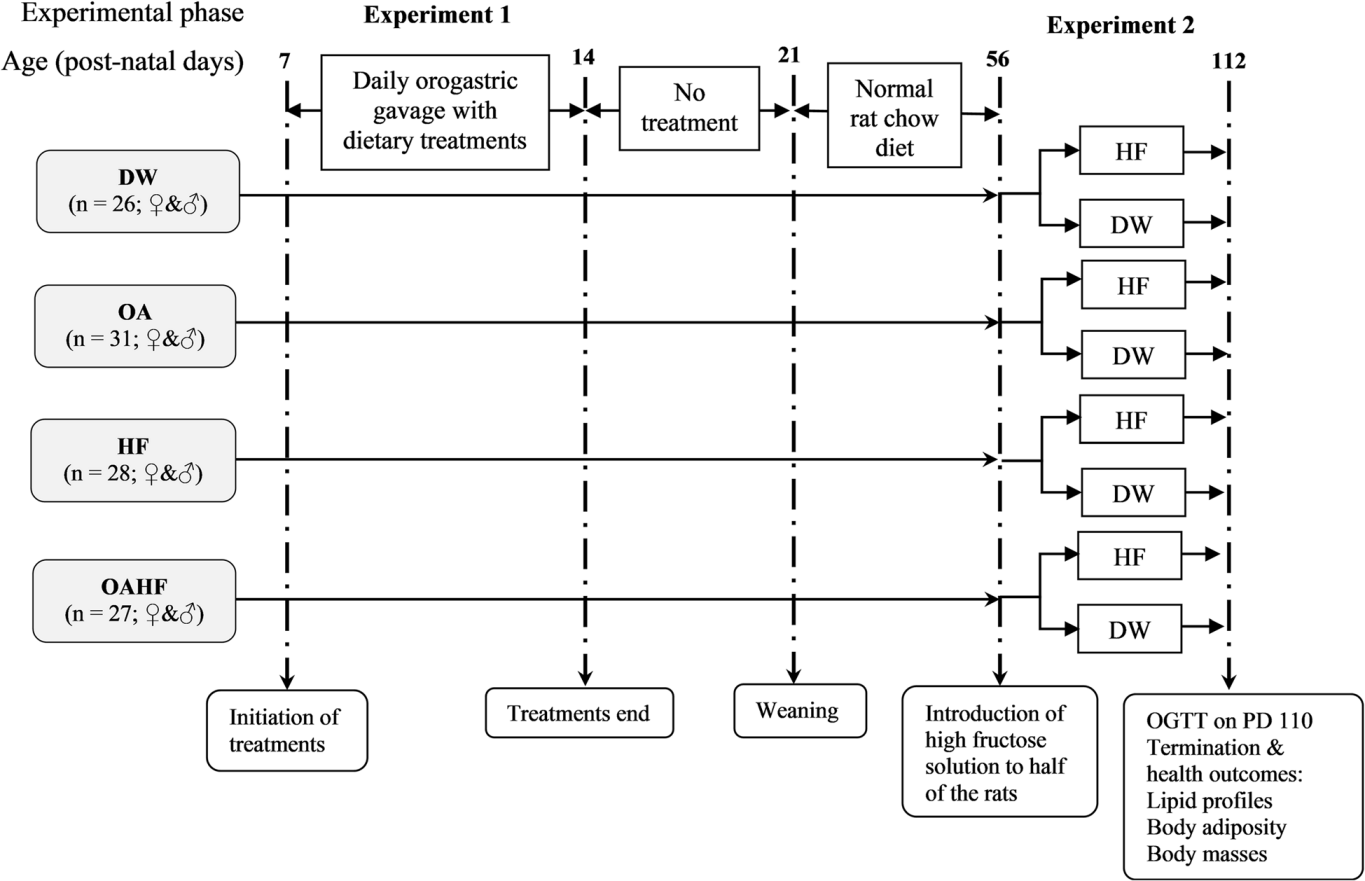
Schematic flow diagram showing the experimental groups, stages of development, sequence and timing of interventions and measurements for the experimental study. **DW** = gavaged daily with 10 mL/kg body mass of distilled water with 0.5% (*v*/v) dimethyl sulphoxide in the neonatal phase (*n* = 26; ♀&♂); **OA** = gavaged daily with 10 mL/kg body mass of oleanolic acid (60 mg/kg) in the neonatal phase (*n* = 31; ♀&♂); **HF** = gavaged daily with 10 mL/kg of 20% (*w*/*v*) fructose solution in the neonatal phase (*n* = 28; ♀&♂); **OAHF** = gavaged daily with 10 mL/kg body mass of oleanolic acid (60 mg/kg) and 20% (w/v) fructose solution in the neonatal period (*n* = 27; ♀&♂); OGGT = oral glucose tolerance test; GIT = gastrointestinal tract; PD = post-natal day; ♀ = female rats; ♂ = male rats

One hundred and twelve male and female pups were used in this interventional study which was conducted in two experimental phases. The timelines and group allocations for the rats are summarised in Fig. 1. The dams with their pups were received on PD3. All rats were acclimatised for 4 days from PD4 up to the commencement of experimental treatment on PD7. During the first experimental phase (PD7-PD14), the first nutritional intervention was introduced in order to induce neonatal programming. The pups were weighed daily to monitor growth performance and in order to adjust treatments so as ensure the desired dosage per body mass. The pups were then randomly assigned to one of four dietary treatment groups:

i). *Control group (DW)* – received distilled water with dimethyl sulphoxide (DMSO) solution (0.5% *v*/v). The DMSO was used as a vehicle control to dissolve the OA for the other groups (*n* = 26; 13 males and 13 females).
ii). *Oleanolic acid (OA) group* – received oleanolic acid (60 mg/kg body mass) reconstituted in DMSO (0.5% *v*/v) (*n* = 31; 15 males and 16 females).
iii). *High fructose solution (HF) group* – received 20% fructose solution (*w*/*v*) reconstituted in DMSO (0.5% *v*/v) (*n* = 28; 13 males and 15 females).
iv). *Oleanolic acid and high fructose solution (OAHF) group* – received oleanolic acid (60 mg/kg body mass) and 20% fructose solution (w/v) reconstituted in DMSO (0.5% v/v) (*n* = 27; 14 males and 13 females).

All treatments in the first experimental phase (Fig. 1) were administered once a day (between 09:00–10:00), for seven consecutive days (PD7 to PD13), at a volume of 10 mL/kg body mass via orogastric gavage using an orogastric tube attached to a 1 mL syringe. After treatments in the first experimental phase, pups were allowed to continue nursing with their dams from PD14 to PD20 until they were weaned on PD21. The dams were returned to stock and the pups were housed individually in acrylic cages where they ad libitum access to SRC and plain drinking tap water until PD55.

In the second experimental phase (PD56 up to PD112), the rats in each of the four experimental treatment groups received ad libitum access to SRC, however, half the number of male and female rats (*n* = 56) in each group received either plain drinking water whilst the other half received 20% (*w*/*v*) fructose solution as drinking fluid. Fresh fructose solution and water were provided every 2 days. Fructose was given in the second phase, as a secondary dietary insult in order to induce health outcomes associated with MetS [62, 63].

In the current study, rats were subjected to either a single or double hit according to the single or double hit hypothesis [64]. The single hit was either an early neonatal administration of fructose (20% *w*/*v*) or the provision of fructose in drinking water (20% w/v) later in adult life. The double hit was characterised by two dietary interventions; a fructose hit in early-life (first-hit) which would predispose the rats to the onset of metabolic derangements followed by another dietary fructose intervention in later life (the second-hit).

### Oral glucose tolerance test

At the end of the 16 week study period (PD110), an oral glucose tolerance test (OGTT) was performed after an overnight fast. Briefly, fasting blood glucose was determined and then each rat was administered with a dose of 2 g/kg body mass of sterile 50% (*w*/*v*) D-glucose solution (Sigma, Johannesburg, South Africa) via orogastric gavage [65]. Thereafter blood glucose concentrations were measured at *T* = 15, 30, 60, 120 and 180 min using a calibrated glucometer (Contour Plus® glucometer, Bayer (Pty) Ltd., Johannesburg, South Africa). The blood samples were collected following a sterile pin prick of the distal tail vein. The incremental changes in blood glucose after administration of the glucose load were expressed as an area under the curve (AUC) from the time when the fasting blood was drawn (*T* = 0) until 180 min post-load blood sampling [66].

### Terminal procedures

After the OGTT, the rats were placed back onto their respective dietary treatments for a further 48 h. Thereafter, the animals were fasted overnight and their fasting blood glucose levels measured using the calibrated glucometer (Contour Plus® glucometer, Bayer (Pty) Ltd., Johannesburg, South Africa). The rats were then euthanased by an overdose of intra-peritoneally injected sodium pentobarbital (200 mg/kg body mass; Eutha-naze®, Bayer Corporation, Johannesburg, South Africa).

#### Blood and adipose tissue sample collection

Following euthanasia of the rats, blood was collected via cardiac puncture using 21G needles and 10 ml syringes and transferred into heparinised blood collection tubes (BD Vacutainer® Systems, Meylan Cedex, France). The blood samples were centrifuged for 15 min at 5000×*g* at 20 °C (Sorvall RT® 6000B centrifuge, Rockville, USA). The collected plasma samples were stored at − 20 °C for the analysis of insulin and cholesterol.

The abdomen was opened via a midline incision and the visceral (and epipidymal in males) fat pads were dissected out and weighed. The left tibia was dissected from the hindlimb of each rat, de-fleshed, dried in an oven (50 °C for 5 days) and the length measured. The length of the tibia, which is less prone to acute variation compared to body mass, was used to calculate the relative masses of visceral and epididymal fat.

#### Determination of circulating cholesterol and triglyceride concentrations

The plasma concentrations of triglycerides were measured using a calibrated triglyceride metre (Accutrend®, Roche, Mannheim, Germany) in accordance with the manufacturer’s instructions. Plasma cholesterol was measured using a calibrated automatic biochemical analyzer (IDEXX VetTest™ Clinical Chemistry Analyser, IDEXX Laboratories Inc., Westbrook, ME, USA) as per the manufacturer’s instructions.

### Measurement of plasma insulin and calculation of the homeostatic model assessment of insulin resistance (HOMA-IR)

Plasma concentrations of insulin were measured using an enzyme-linked immunosorbent assay (ELISA) kit for rats (Elabscience ®, Rat INS ELISA kit, Wuhan, China) following the manufacturer’s instructions. Insulin resistance was calculated by means of the homeostatic model assessment index (HOMA-IR) using the relationship between the fasting blood glucose and insulin levels, according to the following formula:

HOMA-IR = Insulin (μU/mL) × Blood glucose (mM)/22.5 [67]. Plasma levels of insulin were converted from ng/g to μU/mL for the calculation of HOMA-IR.

### Statistical analyses

Data were expressed as mean ± standard deviation and analysed using GraphPad Prism for Windows Version 7.0 (GraphPad Software Inc., San Diego, USA). The baseline fasting glucose levels were subtracted from all other glucose values for each animal and the total area under the curve (AUC) for the OGTT was calculated by the trapezoidal method to assess glucose tolerance [65]. A two-way repeated measures analysis of variance (ANOVA), with Bonferroni *post-hoc* test, was used to analyse terminal body mass with day as a within-subjects factor and treatment as a between-subjects factor. A one-way ANOVA with Bonferroni *post-hoc* test was used to compare the means for all parameters measured from different treatment groups. The level of significance acceptable was *P ≤* 0.05.

## Results

### The effect of neonatal oral administration of oleanolic acid on weaning and terminal body masses of fructose-fed male and female rats

**Fig. 2:**
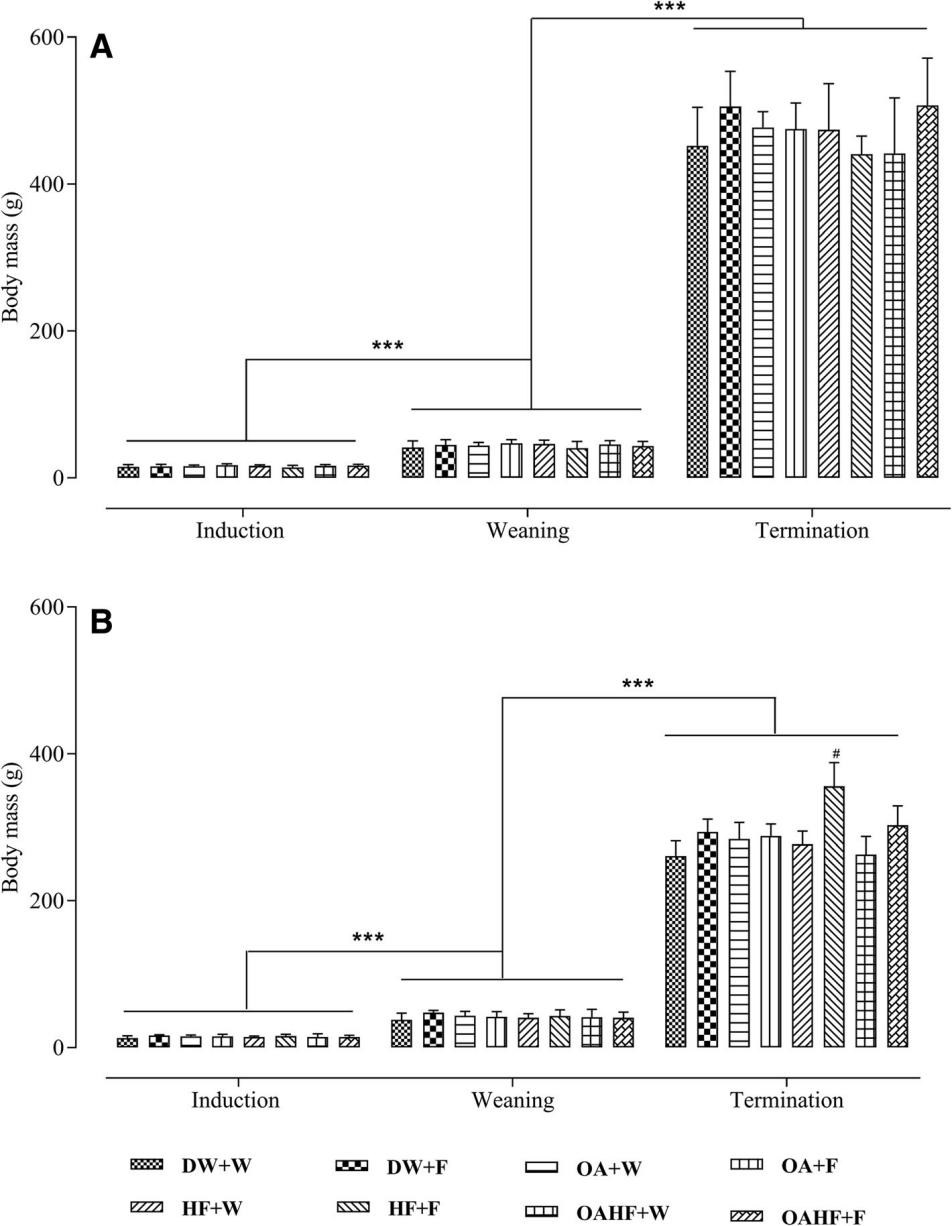
The effect of neonatal oral administration of oleanolic acid on weaning and terminal body masses of male (**a**) and female (**b**) rats fed a high fructose diet. All data presented as mean ± standard deviation. ^***^ Significant increase in body mass from induction to weaning and from weaning to termination (*P* < 0.005). ^#^ Significant increase in terminal body mass for female rats receiving a double hit of fructose (HF + F) compared to all of the other treatment groups (*P* < 0.005). **DW + W** = gavaged daily with 10 mL/kg body mass of distilled water with DMSO (0.5% v/v) in the neonatal period followed by ad libitum access to plain drinking water post-weaning and throughout adulthood (male = 7; female = 7); **DW + F** = gavaged daily with 10 mL/kg body mass of distilled water with DMSO (0.5% *v*/v) in the neonatal period followed by ad libitum access to 20% (*w*/*v*) fructose solution as drinking fluid in adulthood (male = 6; female = 6); **OA + W** = gavaged daily with 60 mg/kg body mass oleanolic acid in the neonatal period followed by ad libitum access to plain drinking water post-weaning and throughout adulthood (male = 8; female = 8); **OA + F** = gavaged with 60 mg/kg body mass oleanolic acid in the neonatal period followed by ad libitum access to 20% (*w*/*v*) fructose as drinking fluid in adulthood (male = 7; female = 8); **HF + W** = gavaged daily with 10 mL/kg body mass 20% (w/v) fructose solution in the neonatal period followed by ad libitum access to plain drinking water post-weaning and throughout adulthood (male = 6; female = 7); **HF + F** = gavaged daily with 10 mL/kg body mass 20% (*w*/*v*) fructose solution in the neonatal period followed by ad libitum access to 20% (w/v) fructose as drinking fluid in adulthood (male = 7; female = 8); **OAHF + W** = gavaged daily with 10 mL/kg body mass of a combination of oleanolic acid (60 mg/kg) and 20% (*w*/*v*) fructose solution in the neonatal period followed by ad libitum access to plain drinking water post-weaning and throughout adulthood (male = 7; female = 6); **OAHF + F** = gavaged daily with 10 mL/kg body mass of a combination of oleanolic acid (60 mg/kg) and 20% (*w*/*v*) fructose solution in the neonatal period followed by ad libitum access to 20% (w/v) fructose solution as drinking fluid in adulthood (male = 7; female = 7)

The induction body masses in male rats were similar across all experimental treatment groups (*P* > 0.05; Fig. 2a). There was a significant increase in body masses of male rats in all experimental treatment groups from induction to weaning and from weaning up to termination (*P* < 0.05; Fig. 2a). There were no significant differences in terminal body masses from all experimental treatment groups (*P* > 0.05; Fig. 2a).

In female rats, induction body masses were also similar across all experimental treatment groups (*P* > 0.05; Fig. 2b). Body masses of female rats from all treatment groups increased significantly from induction to weaning and from weaning up to termination (*P* < 0.05; Fig. 2b). A double hit of fructose, (neonatally and in adulthood, HF + F) caused a significant increase in terminal body mass compared to other experimental groups (*P* < 0.05; Fig. 2b). Oral administration of OA in the neonatal period (OAHF+F) prevented the increase in terminal body mass recorded in the rats that received the double hit of fructose (HF + F) (*P* < 0.05; Fig. 2b). Neonatal administration of OA therefore prevented the increase in terminal body mass due to a double hit with fructose in female rats.

### The effect of neonatal oral administration of oleanolic acid on glycaemic control in fructose-fed male and female rats

#### Oral glucose tolerance test (OGTT)

**Fig. 3:**
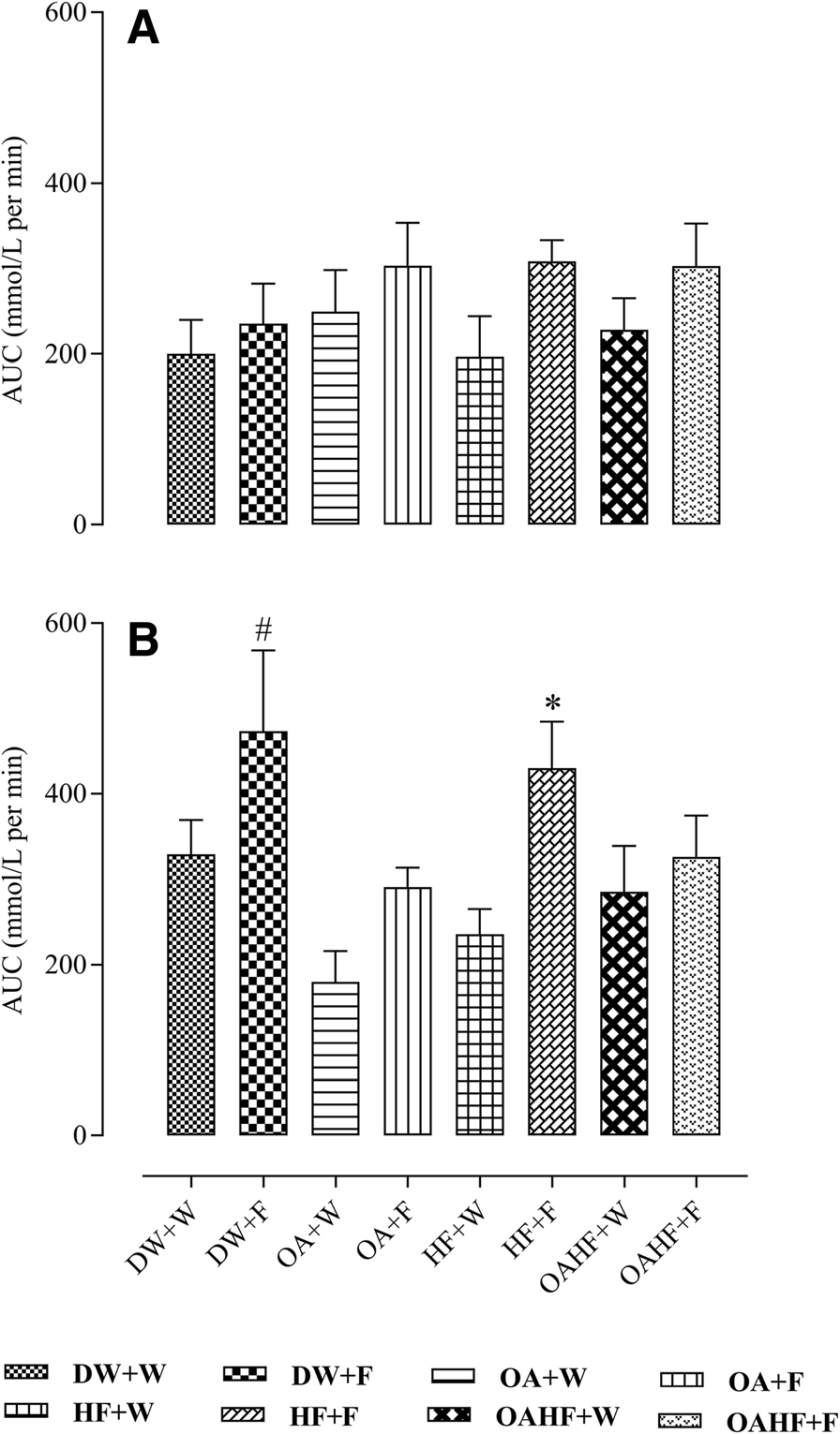
Effect of neonatal oral administration of oleanolic acid and fructose on the total area under the curve of the oral glucose tolerance test of male (**a**) and female (**b**) rats fed a high fructose diet. All data presented as mean ± standard deviation. ^#*^Significant increase in total AUC (*P*<0.05) compared to other experimental treatment groups. **DW + W** = gavaged daily with 10 mL/kg body mass of distilled water with DMSO (0.5% *v*/v) in the neonatal period followed by ad libitum access to plain drinking water post-weaning and throughout adulthood (male = 7; female = 7); **DW + F** = gavaged daily with 10 mL/kg body mass of distilled water with DMSO (0.5% v/v) in the neonatal period followed by ad libitum access to 20% (*w*/*v*) fructose solution as drinking fluid in adulthood (male = 6; female = 6); **OA + W** = gavaged daily with 60 mg/kg body mass oleanolic acid in the neonatal period followed by ad libitum access to plain drinking water post-weaning and throughout adulthood (male = 8; female = 8); **OA + F** = gavaged with 60 mg/kg body mass oleanolic acid in the neonatal period followed by ad libitum access to 20% (w/v) fructose as drinking fluid in adulthood (male = 7; female = 8); **HF + W** = gavaged daily with 10 mL/kg body mass 20% (w/v) fructose solution in the neonatal period followed by ad libitum access to plain drinking water post-weaning and throughout adulthood (male = 6; female = 7); **HF + F** = gavaged daily with 10 mL/kg body mass 20% (w/v) fructose solution in the neonatal period followed by ad libitum access to 20% (w/v) fructose as drinking fluid in adulthood (male = 7; female = 8); **OAHF + W** = gavaged daily with 10 mL/kg body mass of a combination of oleanolic acid (60 mg/kg) and 20% (w/v) fructose solution in the neonatal period followed by ad libitum access to plain drinking water post-weaning and throughout adulthood (male = 7; female = 6); **OAHF + F** = gavaged daily with 10 mL/kg body mass of a combination of oleanolic acid (60 mg/kg) and 20% (w/v) fructose solution in the neonatal period followed by ad libitum access to 20% (w/v) fructose solution as drinking fluid in adulthood (male = 7; female = 7)

There was no significant difference in the total area under the curve (AUC) of oral glucose tolerance test (OGTT) for male rats across all experimental treatment groups (*P* > 0.05; Fig. 3a).

In female rats, fructose consumption either late in adulthood (DW + F) or as a double hit early in the neonatal period and late in adulthood (HF + F) resulted in up to 37% increase in the total AUC compared with other experimental treatment groups (*P <* 0.05; Fig. 3b). Neonatal administration of OA prevented the increase in AUC induced by the late single hit of fructose (OA + F) and a double hit (OAHF+F) fructose effects (*P* < 0.05; Fig. 3b). There were no significant differences in the total AUC in rats that received neonatal OA and the control group that did not receive fructose (DW + W) (*P* > 0.05).

#### Fasting blood glucose, insulin and homeostatic model assessment of insulin resistance (HOMA-IR)

In male rats, fructose consumption either late in adulthood (DW + F) or a double hit (HF + F) resulted in an increase in fasting levels of glucose and the HOMA-IR compared to other treatment groups (*P* < 0.05; Table 1). However, there were no significant differences in fasting levels of insulin in male rats across all treatment groups (*P* > 0.05; Table 1). There were no significant differences in the insulin levels in rats that received neonatal OA and the control group that did not receive fructose (DW + W) (*P* > 0.05). Oral administration of OA in the neonatal period prevented the increase in fasting glucose and HOMA-IR observed as a result of the double hit with fructose (OA + F vs OAHF+F) *P* > 0.001; Table 1).

**Table 1:** Effect of neonatal oral administration of oleanolic acid and or fructose on fasting blood glucose and plasma insulin concentration and HOMA-IR index in male rats fed a high fructose diet

Parameter	DW + W	DW + F	OA + W	OA + F	HF + W	HF + F	OAHF + W	OAHF + F
Glucose (mmol/L)	4.9 ± 0.7	5.3 ± 0.3	4.5 ± 0.5	4.7 ± 0.6	4.5 ± 0.4	^a^6.3 ± 0.7	4.8 ± 0.5	4.5 ± 1.1
Insulin (μU/mL)	45.3 ± 10.5	53.2 ± 10.2	44.6 ± 9.4	51.4 ± 8.7	39.8 ± 5.2	56.9 ± 7.9	41.9 ± 10.2	46.5 ± 16.5
HOMA-IR	0.24 ± 0.06	^b^0.33 ± 0.06	0.23 ± 0.05	0.27 ± 0.07	0.18 ± 0.03	^c^0.35 ± 0.12	0.22 ± 0.05	0.22 ± 0.07

All data presented as mean ± standard deviation

^abc^ Significant increase in glucose and abc HOMA-IR (*P* < 0.05) for groups receiving fructose late (DW + F) and a double hit (neonatally and in adulthood, HF + F) compared with other treatment groups. **DW + W** = gavaged daily with 10 mL/kg body mass of distilled water with DMSO (0.5% v/v) in the neonatal period followed by ad libitum access to plain drinking water post-weaning and throughout adulthood (male = 7; female = 7); **DW + F** = gavaged daily with 10 mL/kg body mass of distilled water with DMSO (0.5% v/v) in the neonatal period followed ad libitum access to 20% (w/v) fructose solution as drinking fluid in adulthood (male = 6; female = 6); **OA + W** = gavaged daily with 60 mg/kg body mass oleanolic acid in the neonatal period followed by ad libitum access to plain drinking water post-weaning and throughout adulthood (male = 8; female = 8); **OA + F** = gavaged with 60 mg/kg body mass oleanolic acid in the neonatal period followed by ad libitum access to 20% (w/v) fructose as drinking fluid in adulthood (male = 7; female = 8); **HF + W** = gavaged daily with 10 mL/kg body mass 20% (w/v) fructose solution in the neonatal period followed by ad libitum access to plain drinking water post-weaning and throughout adulthood (male = 6; female = 7); **HF + F** = gavaged daily with 10 mL/kg body mass 20% (w/v) fructose solution in the neonatal period followed by ad libitum access to 20% (w/v) fructose as drinking fluid in adulthood (male = 7; female = 8); **OAHF + W** = gavaged daily with 10 mL/kg body mass of a combination of oleanolic acid (60 mg/kg) and 20% (w/v) fructose solution in the neonatal period followed by ad libitum access to plain drinking water post-weaning and throughout adulthood (male = 7; female = 6); **OAHF + F** = gavaged daily with 10 mL/kg body mass of a combination of oleanolic acid (60 mg/kg) and 20% (w/v) fructose solution in the neonatal period followed by ad libitum access to 20% (w/v) fructose solution as drinking fluid in adulthood (male = 7; female = 7)

In female rats, a double hit of fructose (early in the neonatal period and late in adulthood: HF + F) resulted in an increase in fasting levels of glucose and HOMA-IR compared with other experimental treatment groups (*P* < 0.05; Table 2). Neonatal oral OA administration with the double hit fructose (OAHF+F) prevented the effects on glucose levels and HOMA-IR observed in the rats that had the double hit of fructose without any other intervention (HF + F) (*P* < 0.05; Table 2). There were no significant differences in the fasting levels of insulin across all treatment groups in female rats (*P* > 0.05; Table 2).

**Table 2:** Effect of neonatal oral administration of oleanolic acid and fructose on fasting blood glucose and plasma insulin concentration and HOMA-IR index in female rats fed a high fructose diet

Parameter	DW + W	DW + F	OA + W	OA + F	HF + W	HF + F	OAHF + W	OAHF + F
Glucose (mmol/L)	4.2 ± 0.3	4.6 ± 0.8	4.8 ± 0.5	4.5 ± 0.6	4.2 ± 0.6	^a^6.2 ± 0.7	4.7 ± 0.9	4.1 ± 0.3
Insulin (μU/mL)	42.2 ± 9.5	45.1 ± 13.1	41.6 ± 12.6	48.1 ± 11.2	43.8 ± 6.3	55.7.0 ± 11.5	46.4 ± 5.2	46.3 ± 6.4
HOMA-IR	0.19 ± 0.05	0.30 ± 0.08	0.22 ± 0.06	0.22 ± 0.07	0.20 ± 0.03	^b^0.38 ± 0.07	0.24 ± 0.05	0.22 ± 0.04

All data presented as mean ± standard deviation

^ab^ Significant increase in glucose and HOMA-IR (*P* < 0.05) for groups receiving a double hit (neonatally and in adulthood, HF + F) compared with other treatment groups. **DW + W** = gavaged daily with 10 mL/kg body mass of distilled water with DMSO (0.5% v/v) in the neonatal period followed by ad libitum access to plain drinking water post-weaning and throughout adulthood (male = 7; female = 7); **DW + F** = gavaged daily with 10 mL/kg body mass of distilled water with DMSO (0.5% v/v) in the neonatal period followed ad libitum access to 20% (w/v) fructose solution as drinking fluid in adulthood (male = 6; female = 6); **OA + W** = gavaged daily with 60 mg/kg body mass oleanolic acid in the neonatal period followed by ad libitum access to plain drinking water post-weaning and throughout adulthood (male = 8; female = 8); **OA + F** = gavaged with 60 mg/kg body mass oleanolic acid in the neonatal period followed by ad libitum access to 20% (w/v) fructose as drinking fluid in adulthood (male = 7; female = 8); **HF + W** = gavaged daily with 10 mL/kg body mass 20% (w/v) fructose solution in the neonatal period followed by ad libitum access to plain drinking water post-weaning and throughout adulthood (male = 6; female = 7); **HF + F** = gavaged daily with 10 mL/kg body mass 20% (w/v) fructose solution in the neonatal period followed by ad libitum access to 20% (w/v) fructose as drinking fluid in adulthood (male = 7; female = 8); **OAHF + W** = gavaged daily with 10 mL/kg body mass of a combination of oleanolic acid (60 mg/kg) and 20% (w/v) fructose solution in the neonatal period followed by ad libitum access to plain drinking water post-weaning and throughout adulthood (male = 7; female = 6); **OAHF + F** = gavaged daily with 10 mL/kg body mass of a combination of oleanolic acid (60 mg/kg) and 20% (w/v) fructose solution in the neonatal period followed by ad libitum access to 20% (w/v) fructose solution as drinking fluid in adulthood (male = 7; female = 7)

All data presented as mean ± standard deviation. ^abc^Significant increase in glucose and HOMA-IR (*P* < 0.05) for groups receiving fructose late (DW + F) and a double hit (neonatally and in adulthood, HF + F) compared with other treatment groups. **DW + W** = gavaged daily with 10 mL/kg body mass of distilled water with DMSO (0.5% v/v) in the neonatal period followed by ad libitum access to plain drinking water post-weaning and throughout adulthood (male = 7; female = 7); **DW + F** = gavaged daily with 10 mL/kg body mass of distilled water with DMSO (0.5% *v*/v) in the neonatal period followed ad libitum access to 20% (*w*/*v*) fructose solution as drinking fluid in adulthood (male = 6; female = 6); **OA + W** = gavaged daily with 60 mg/kg body mass oleanolic acid in the neonatal period followed by ad libitum access to plain drinking water post-weaning and throughout adulthood (male = 8; female = 8); **OA + F** = gavaged with 60 mg/kg body mass oleanolic acid in the neonatal period followed by ad libitum access to 20% (*w*/*v*) fructose as drinking fluid in adulthood (male = 7; female = 8); **HF + W** = gavaged daily with 10 mL/kg body mass 20% (*w*/*v*) fructose solution in the neonatal period followed by ad libitum access to plain drinking water post-weaning and throughout adulthood (male = 6; female = 7); **HF + F** = gavaged daily with 10 mL/kg body mass 20% (w/v) fructose solution in the neonatal period followed by ad libitum access to 20% (w/v) fructose as drinking fluid in adulthood (male = 7; female = 8); **OAHF + W** = gavaged daily with 10 mL/kg body mass of a combination of oleanolic acid (60 mg/kg) and 20% (w/v) fructose solution in the neonatal period followed by ad libitum access to plain drinking water post-weaning and throughout adulthood (male = 7; female = 6); **OAHF + F** = gavaged daily with 10 mL/kg body mass of a combination of oleanolic acid (60 mg/kg) and 20% (w/v) fructose solution in the neonatal period followed by ad libitum access to 20% (w/v) fructose solution as drinking fluid in adulthood (male = 7; female = 7).

### The effect of neonatal oral administration of oleanolic acid on **v**isceral and epididymal fat masses in fructose-fed rats

**Fig. 4:**
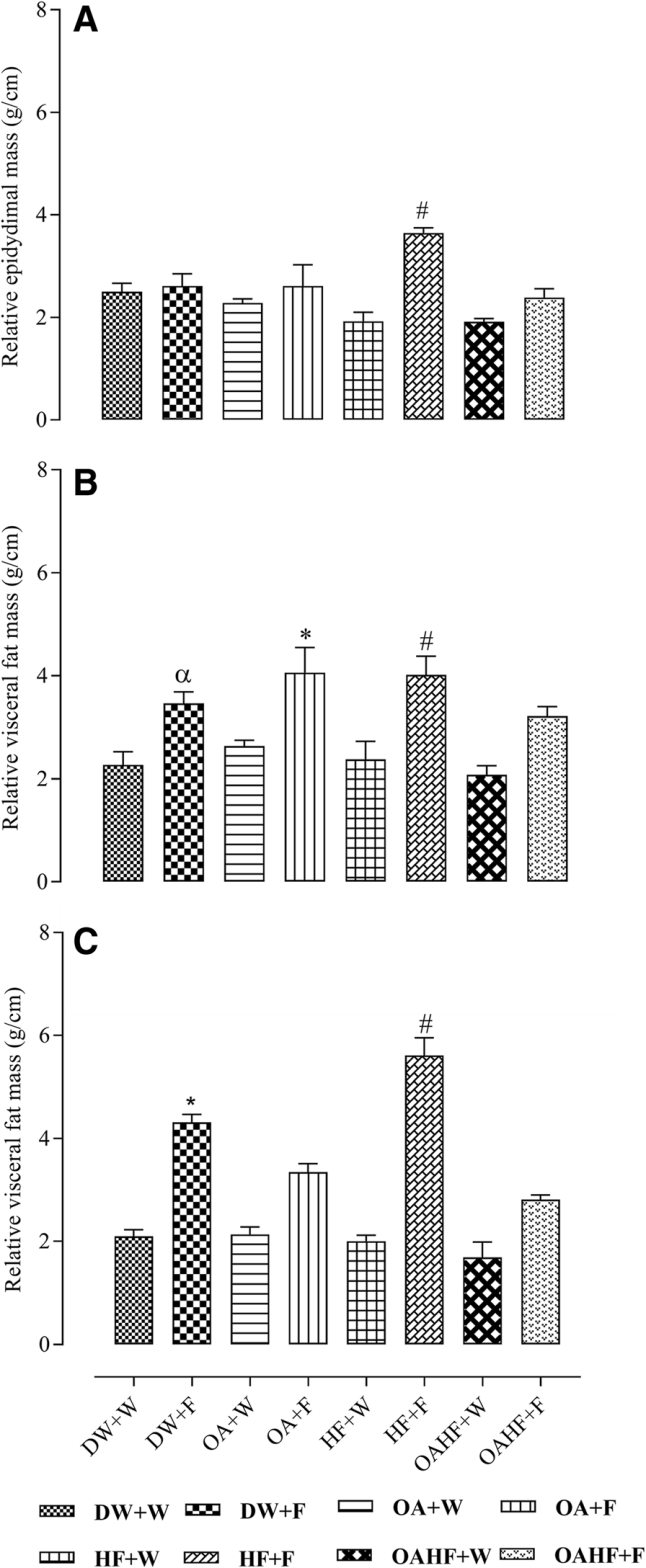
Effect of neonatal oral administration of oleanolic acid and fructose on relative epididymal fat mass (**a**), and visceral fat mass in male (**b**) and female (**c**) rats fed a high fructose diet. All data presented as mean ± standard deviation. ^α#*^Significant increase in relative epididymal fat pad masses for male rats receiving a double fructose hit (HF + F) and relative visceral fat masses of male (2.6 ± 0.6 g/cm) and female (4.3 ± 0.4) rats receiving a single late fructose hit (DW + F) or male (3.7 ± 0.3 g/cm) and female (5.6 ± 0.9 g/cm) receiving a double fructose hit (as neonates and later in adulthood) HF + F (*P* < 0.05). **DW + W** = gavaged daily with 10 mL/kg body mass of distilled water with DMSO (0.5% v/v) in the neonatal period followed by ad libitum access to plain drinking water post-weaning and throughout adulthood (male = 7; female = 7); **DW + F** = gavaged daily with 10 mL/kg body mass of distilled water with DMSO (0.5% v/v) in the neonatal period followed by ad libitum access to 20% (w/v) fructose solution as drinking fluid in adulthood (male = 6; female = 6); **OA + W** = gavaged daily with 60 mg/kg body mass oleanolic acid in the neonatal period followed by ad libitum access to plain drinking water post-weaning and throughout adulthood (male = 8; female = 8); **OA + F** = gavaged with 60 mg/kg body mass oleanolic acid in the neonatal period followed by ad libitum access to 20% (w/v) fructose as drinking fluid in adulthood (male = 7; female = 8); **HF + W** = gavaged daily with 10 mL/kg body mass 20% (w/v) fructose solution in the neonatal period followed by ad libitum access to plain drinking water post-weaning and throughout adulthood (male = 6; female = 7); **HF + F** = gavaged daily with 10 mL/kg body mass 20% (w/v) fructose solution in the neonatal period followed by ad libitum access to 20% (w/v) fructose as drinking fluid in adulthood (male = 7; female = 8); **OAHF + W** = gavaged daily with 10 mL/kg body mass of a combination of oleanolic acid (60 mg/kg) and 20% (w/v) fructose solution in the neonatal period followed by ad libitum access to plain drinking water post-weaning and throughout adulthood (male = 7; female = 6); **OAHF + F** = gavaged daily with 10 mL/kg body mass of a combination of oleanolic acid (60 mg/kg) and 20% (w/v) fructose solution in the neonatal period followed by ad libitum access to 20% (w/v) fructose solution as drinking fluid in adulthood (male = 7; female = 7)

Male rats that received fructose either late in adulthood, with or without neonatal OA (DW + F and OA + F) or as a double hit (neonatally and late in adulthood, HF + F) had up to 40% increase in the mass of the relative epididymal fat pads (*P* < 0.05; Fig. 4a). A double hit with fructose (early in the neonatal period and late in adulthood, HF + F) caused up to 40% increase in relative visceral fat mass (adjusted to relative tibial length) compared to the other experimental treatment groups (*P* < 0.05; Fig. 4b). Rats which received neonatal oral administration of OA and the double hit of fructose (OAHF+F) had significantly lower measures of adiposity than the rats which had the double hit of fructose without any other treatment intervention (HF + F) (*P* < 0.05; Fig. 4b), but there was no significant difference when compared to the rats that had the late hit with fructose (OA + F) (*P* > 0.05; Fig. 4b).

In female rats, fructose consumption either late in adulthood (DW + F) or as a double hit (early neonatally and late in adulthood, HF + F) resulted in up to 26% and 65% respectively increase in relative visceral fat masses compared to the other experimental groups (*P* < 0.05; Fig. 4c). Neonatal oral OA administration prevented the late single hit (OA + F group) and the double hit (OAHF+F group) (*P* < 0.05; Fig. 4c) fructose effects on visceral fat mass. No significant differences were observed between the group that received neonatal OA and the control group which did not receive fructose (DW + W) later in adulthood (*P* > 0.05; Fig. 4c).

### The effect of neonatal oral administration of oleanolic acid on the concentration of circulating cholesterol and triglycerides in fructose-fed male and female rats

There were no significant differences in fasting triglyceride and cholesterol levels of the male rats across all experimental dietary treatments (*P* > 0.05; Table 3).

**Table 3:** Effect of neonatal oral administration of oleanolic acid and fructose on circulating triglycerides and cholesterol in male rats fed a high fructose diet

Parameter	DW + W	DW + F	OA + W	OA + F	HF + W	HF + F	OAHF + W	OAHF + F
TG (mmol/L)	1.5 ± 0.2	2.2 ± 0.7	1.5 ± 0.3	1.9 ± 0.4	1.5 ± 0.1	2.1 ± 0.5	1.7 ± 0.7	2.1 ± 0.7
CHOL (mmol/L)	3.7 ± 0.7	3.9 ± 0.4	3.7 ± 0.7	4.1 ± 0.5	3.2 ± 0.7	3.4 ± 0.4	3.8 ± 0.6	3.2 ± 0.9

The levels of circulating triglycerides in female rats that received only a late fructose hit (DW + F) and those that received the double hit with fructose (in the neonatal period and in adulthood, HF + F) significantly increased by up to 65% than rats from the other experimental treatment groups (*P* < 0.05; Table 4). There were no significant differences in fasting cholesterol levels of the female rats across all experimental dietary treatments (*P* > 0.05; Table 4). Oral administration of OA in the neonatal period prevented the increase in the levels of triglycerides observed as a result of either a late single hit or a double hit with fructose (OA + F vs DW + F; OAHF+F vs HF + F respectively; *P* < 0.05; Table 4).

**Table 4:** Effect of neonatal oral administration of oleanolic acid and fructose on circulating triglycerides and cholesterol in female rats fed a high fructose diet

Parameter	DW + W	DW + F	OA + W	OA + F	HF + W	HF + F	OAHF + W	OAHF + F
TG (mmol/L)	1.5 ± 0.3	^a^2.8 ± 0.6	1.5 ± 0.2	1.7 ± 0.3	1.6 ± 0.2	^b^2.7 ± 0.4	1.4 ± 0.3	1.6 ± 0.4
CHOL (mmol/L)	2.9 ± 1.3	4.1 ± 1.0	3.1 ± 0.4	3.9 ± 0.8	3.2 ± 0.4	4.0 ± 0.6	2.6 ± 1.7	3.2 ± 1.1

All data presented as mean ± standard deviation. ^ab^Significant increase in TG levels in rats that received a late fructose hit (DW + F) and those that received fructose neonatally and as adults (HF + F) compared rats from other experimental treatment groups (*P* < 0.05). **DW + W** = gavaged daily with 10 mL/kg body mass of distilled water with DMSO (0.5% v/v) in the neonatal period followed by ad libitum access to plain drinking water post-weaning and throughout adulthood (male = 7; female = 7); **DW + F** = gavaged daily with 10 mL/kg body mass of distilled water with DMSO (0.5% *v*/v) in the neonatal period followed ad libitum access to 20% (*w*/*v*) fructose solution as drinking fluid in adulthood (male = 6; female = 6); **OA + W** = gavaged daily with 60 mg/kg body mass oleanolic acid in the neonatal period followed by ad libitum access to plain drinking water post-weaning and throughout adulthood (male = 8; female = 8); **OA + F** = gavaged with 60 mg/kg body mass oleanolic acid in the neonatal period followed by ad libitum access to 20% (*w*/*v*) fructose as drinking fluid in adulthood (male = 7; female = 8); **HF + W** = gavaged daily with 10 mL/kg body mass 20% (w/v) fructose solution in the neonatal period followed by ad libitum access to plain drinking water post-weaning and throughout adulthood (male = 6; female = 7); **HF + F** = gavaged daily with 10 ml/kg body mass 20% (w/v) fructose solution in the neonatal period followed by ad libitum access to 20% (w/v) fructose as drinking fluid in adulthood (male = 7; female = 8); **OAHF + W** = gavaged daily with 10 mL/kg body mass of a combination of oleanolic acid (60 mg/kg) and 20% (w/v) fructose solution in the neonatal period followed by ad libitum access to plain drinking water post-weaning and throughout adulthood (male = 7; female = 6); **OAHF + F** = gavaged daily with 10 mL/kg body mass of a combination of oleanolic acid (60 mg/kg) and 20% (w/v) fructose solution in the neonatal period followed by ad libitum access to 20% (w/v) fructose solution as drinking fluid in adulthood (male = 7; female = 7). TG = triglycerides; CHOL = cholesterol.

## Discussion

In this study, we sought to investigate the potential protective effect of neonatal oral administration of oleanolic acid (OA) against the subsequent development of health outcomes associated with metabolic dysfunction induced by the consumption of fructose in adult male and female rats. Fructose caused metabolic derangements in male and female rats, however, this was affected by the timing of the fructose intervention(s). We also noted sex differences in responses to the high fructose diets. We have shown that a double hit of fructose wherein it was administered in the neonatal period followed by a secondary dietary insult in adulthood resulted in the development of several negative health outcomes associated with metabolic dysfunction, namely the significant increases in terminal body mass (females only), visceral fat mass (males and females), serum triglycerides (females only), epididymal fat (males only), fasting plasma glucose (males and females), impaired glucose metabolism (females only), β-cell dysfunction and insulin resistance (males and females). The single late fructose hit in adulthood resulted in impaired glucose metabolism, increased visceral fat pad masses and levels of triglycerides in female but not male rats. Oral administration of OA in the neonatal period successfully attenuated the manifestation of fructose-induced metabolic disorders in both male and female rats.

### The effect of neonatal oral administration of oleanolic acid on terminal body masses in fructose-fed male and female rats

Our findings showed an increase in the body masses of male and female rats across all the experimental treatment groups from induction to weaning and from weaning to termination. There were no differences in terminal body masses of male rats across all treatment groups. However, the female rats that received a double fructose hit had increased terminal body masses, which was not observed in male rats, and was prevented the administration of OA in the neonatal period. Previous studies have shown that consumption of fructose increases energy intake and body weight [68, 69]. In older rats, the excess calories are stored as fat while in young animals the energy can be channelled into the metabolic costs of growth. This may explain the difference in our findings regarding the effects of fructose on body mass when compared with previous research findings. The sex differences in the response to fructose dietary treatments are discussed later. Previous findings in human and animal studies which showed that neonatal consumption of fructose (early single hit) resulted in abnormal body weight gain [70, 71] which is contrary to our findings. The differences could be explained in part by the fructose dosage that was administered and the duration over which the fructose was administered. While increased body mass alone may not represent obesity, the co-existence of other obesogenic variables such as accumulation of visceral fat, increased levels of triglycerides (TG) as we have shown, provides confirmation of the obese status especially in the female rats [71].

The oral administration of OA in the neonatal period attenuated the increase in terminal body masses caused by fructose in female rats. This finding suggests that neonatal interventions with OA prevent excessive body mass gain later in adulthood, as such OA may therefore be used in the neonatal period against excessive body mass gain in adulthood. Oleanolic acid (OA) has been reported to decrease food intake [72], and lower plasma glucose levels in murine models of diabetes mellitus even after the dosage has been discontinued [50]. A limitation of the current study is that feed and fluid intake were not assessed. Nevertheless, the findings from previous studies support the role of the effect of the OA on food intake and suggest that the effect of OA may be long-lasting. The significantly higher body weights recorded in female rats that received a double fructose hit and the visceral fat mass in rats that did not receive OA and those that received OA with high-fructose groups suggest that fructose consumption may have played a role in the effect of OA on plasma glucose and triglyceride levels. The observed effect of OA on body mass could also be due to the role of neonatal programming. Further investigations using murine models are however required to elucidate the specific molecular mechanism(s) through which OA prevents the body mass increases induced by fructose consumption.

### The effect of neonatal oral administration of oleanolic acid on visceral and epididymal fat masses in fructose-fed adult male and female rats

Findings from this study showed that consumption of fructose in adulthood only and when consumed as a double hit (neonatally and in adulthood) resulted in visceral fat (relative to tibial length) accumulation in both male and female rats. However, it was the double hit of fructose that caused an average of approximately 40% increase in epididymal fat mass in male rats compared to the control and other treatment groups. Our findings suggest that the double fructose hit may have programmed the accumulation of epididymal fat in males as well as visceral fat accumulation in both male and female rats. Due to acute fluctuations in body mass as a result of factors such as the hydration status of animals and gut fill, body mass can be a less reliable index of growth or organ masses, than the linear growth of the long bones which is less likely to fluctuate acutely and thus would have less of a variability on the calculation of relative organ masses [73]. Consequently, the use of tibial length was used as a ‘stable’ indicator of linear growth in the calculation of the relative fat masses. While previous studies [71, 74] using a late hit with fructose in adults also show similar findings to ours regarding the late hit, it is notable that the impact of the double hit on obesity was much greater than the single hit. The total adipose tissue accumulation plays an important role in the development of metabolic disorders such as insulin resistance (IR), T2DM and CVDs [75]. However, there are some fat depots that are more associated with the development of metabolic risk factors than others [76]. A human study has shown that in men, the excessive accretion of omental and mesenteric adipose tissue, both part of visceral adipose tissue (VAT), is strongly associated with the development of CVDs and T2DM [77]. In rodents however, gonadal VAT surrounding testis (epididymal fat) and ovaries is regarded as one of the largest depots that contribute to the development of metabolic disorders in rodents on a high-energy cafeteria diet [76]. It is therefore possible that the difference in the epididymal fat and the rest of the visceral fat responses observed in male rats that received a double hit of fructose could be due to epididymal fat being more vulnerable to accumulate than the other visceral fat depots in response to high-fructose diets. Based on recent neonatal programming studies [78, 79] and results from this study, we hypothesise that neonatal fructose consumption may have programed adipose tissue development during the critical period which resulted in the observed visceral (male and female rats) and epididymal fat (male rats) accumulation later in adulthood. The neonatal programming of visceral fat accumulation and lipid metabolism induced by fructose may have caused the development and manifestation of other MetS-associated outcomes in adulthood upon the introduction of a secondary dietary fructose insult as we have already shown with glucose metabolism in this study.

The significantly heavier visceral fat pad masses observed in this study in fructose-fed female, but not in male rats, corroborate previous findings which also reported an increase in visceral fat deposition and a corresponding increase in body mass following excessive consumption of fructose in adulthood [80]. Despite the lack of differences in terminal body masses of the male rats that received either a double hit of fructose or a single late hit, as observed in female rats, there were differences in visceral (both males and females) and epididymal (males only) fat mass. Findings from previous studies also suggest that excessive consumption of fructose increases the levels of plasma triglycerides which may accelerate the accretion of body fat and causing visceral obesity and body mass gain [70, 81], a trend that we have observed in the current study, especially in female rats that received a double hit or a single late fructose hit. There are a number of studies in the literature in which fructose solutions, provided along with a chow diet, increase energy intake and body weight [71] glucose intolerance, as well as plasma insulin and triglyceride levels [68].

Although male and female rats receiving a single late and a double fructose hit exhibited increased visceral fat mass relative to tibial length compared with all other treatment groups, this increase was higher and pronounced in female (an average of approximately 26% single hit and an average of approximately 65% double hit) than in male (an average of approximately 40% double hit) rats. This finding suggests that female offspring were vulnerable to greater fructose-induced adiposity than their male counterparts, a finding that is in line with the greater body mass increase in fructose-fed females (double hit), also previously reported by Bayol S, Simbi B, Bertrand J and Stickland N [82]. Androgens and female hormones, which control several metabolic pathways [83] and are involved in the pathogenesis of metabolic disorders [84] may have contributed to the observed sexual dimorphic differences in obesity observed in this study. Androgens promote cellular glucose uptake and energy utilisation in skeletal muscles and liver [85] thus reducing the tendency to accumulate total body fat in men.

The excessive accumulation of epididymal fat in male rats is linked to the development of infertility [86], due to the disruption of testicular physiology, hormonal regulation and metabolism [87]. The infertility can be passed on to future generations through epigenetic modifications passed by the male gametes [87]. It is interesting to note that neonatal oral administration of OA protected against fructose diet-induced increase in epididymal fat mass in males and visceral fat pad masses caused by either a single late hit or a double fructose hit in both male and female rats. Previous animal studies have shown that OA administration in adulthood exhibited hepatic lipid-lowering effects by decreasing hepatic expression of peroxisome proliferator-activated receptor-γ coactivator-1β (PGC-1β), an important regulator in maintaining hepatic lipid homeostasis, and its associated downstream target genes [60]. However further studies should be done using the current animal model to elucidate the molecular mechanisms underlying the observed visceral and epididymal fat lowering effect of OA administered during the neonatal period.

### The effect of neonatal oral administration of oleanolic acid on glucose tolerance in fructose-fed adult male and female rats

Both a late single hit and a double hit of 20% *w*/*v* fructose solution as drinking fluid caused glucose intolerance (total area under the curve of the OGTT) in the female, but not in male rats. The late single fructose hit resulted in the development of IR or β-cell dysfunction (HOMA-IR) in male but not in female rats. However, the double fructose hit caused IR or β-cell dysfunction and hyperglycaemia in both male and female rats. None of the treatments induced an increase in the levels of insulin in both male and female rats.

The oral glucose tolerance test (OGTT) is an important clinical tool for the characterisation of metabolic phenoype [88]. It is used to diagnose impaired glucose tolerance and as a standardised test of carbohydrate metabolism by assessing the ability to dispose of an oral glucose load over time [88, 89]. Sustained hyperglycaemia (> 120 min) in plasma glucose constitutes impaired glucose tolerance and can be used together with fasting hyperglycaemia to diagnose patients with T2DM [66].

Our findings on glucose tolerance suggest that a double fructose hit adversely affected glucose metabolism by impairing the ability to tolerate a glucose load in female rats and caused IR and possibly pancreatic β-cell dysfunction in both male and female rats. Insulin resistance reported in this study for male and female rats that received a double hit of fructose also corroborates earlier findings by Huynh M, Luiken JJ, Coumans W and Bell RC [80] who also demonstrated the manifestation of IR following a double hit of fructose neonatally and in adulthood. However, results on the levels of insulin in fructose-fed rats from the current study are at variance with the same study by Huynh M, Luiken JJ, Coumans W and Bell RC [80] who reported the development of hyperinsulinaemia in rats that received 10% fructose in the neonatal period and 65% fructose diet in adulthood. The observed variance in levels of insulin can be explained in part by the differences in the quantity and method of fructose administration in adulthood. In the current study, adult rats received 20% *w*/*v* fructose solution as a secondary dietary insult, in contrast to Huynh M, Luiken JJ, Coumans W and Bell RC [80] who gave 65% *w*/w in the feed which possibly provided more calories resulting in hyperinsulinaemia which we did not observe. Although hyperglycaemia alone does not indicate whether there is an insufficiency of insulin secretion [89], it is possible that its development observed in this study following a double hit of fructose in both male and female rats coupled with impaired glucose tolerance and IR suggests that the fructose that was administered in the neonatal period may have been effective in programming the neonatal rats for the development of hyperglycaemia, impaired glucose tolerance and IR or pancreatic β-cell dysfunction later in adulthood after exposure to a secondary dietary insult.

The development of hyperglycaemia, glucose intolerance and IR that was induced by either a single late and double fructose hit was prevented by the neonatal oral administration of OA in male and female rats. These findings expand on previous research that reported the anti-diabetic effects of plant-derived OA in adult rats [49]. Diabetes is characterised by persistent hyperglycaemia, poor glycaemic control and IR [67]. Reactive oxygen species (ROS) have been suspected to play a role in the progression from normal glucose metabolism to impaired glucose tolerance and development of insulin resistance [35]. By upregulating the gene expression of anti-oxidant enzymes, glutathione peroxidase and superoxide dismutase, OA promotes anti-oxidant cellular defences which play a role in its glucose-lowering effects [35]. Previous studies have demonstrated the anti-oxidant and anti-glycative role of OA, as such it is possible that the protective role of OA against the development of dysregulation of glucose metabolism observed in this study following a late single hit or a double fructose hit, could be due to the ability of OA to scavenge for free radicals and enhancing anti-oxidant cellular defence system [58]. The observed improvement of glucose tolerance and IR by OA also suggests that OA may be promoting insulin signal transduction mechanisms and inhibiting poor handling of glucose caused by oxidative stress and IR [35].

OA also protects against oxidative stress-induced IR [35]. Mechanistic studies have shown that triterpenoid compounds such as OA act as hypoglycaemic and anti-obesity agents mainly through (i) reducing the intestinal absorption of glucose; (ii) decreasing endogenous glucose production such as hepatic gluconeogenesis; (iii) increasing insulin sensitivity; (iv) improving lipid metabolism; and (v) promoting body weight loss [90]. In addition to these promising beneficial effects, it is believed that OA and associated triterpenoid protect against diabetes-related comorbidities due to their anti-atherogenic, anti-inflammatory, and anti-oxidant properties [58].

### The effect of neonatal administration of oleanolic acid on circulating triglycerides and cholesterol in fructose-fed adult male and female rats

The late single fructose hit and a double hit of fructose caused an increase in the level of triglycerides (TGs) in female but not in male rats. Contrary to our findings, a study in adult male and female rats has shown that excessive consumption of fructose causes an increase in body weight that is accompanied by an increase in VAT and elevated circulating levels of TGs in rats [71]. The observed hypertriglyceridaemia in female rats following administration of a double fructose hit could be explained in part by the fructose-mediated neonatal programming of lipogenic genes [91, 92] and the increased vulnerability to develop hypertriglyceridaemia in fructose-fed female rats. Although male rats that received a late single fructose hit and a double hit of fructose had increased visceral fat accretion, they neither had increased terminal body weights nor TG levels. Regional differences in adipose tissue distribution which is affected by sex hormones may have resulted in the observed sexual dimorphic differences in circulating TG levels. Fructose is predominantly metabolised in the liver and due to its high lipogenic potential, its excessive consumption is likely to increase the metabolic burden on the liver resulting in the development of hepatic steatosis through de novo lipogenesis (DNL) [71]. The observed increase in the levels of TGs in fructose-fed female animals could also be due to the upregulation of hepatic DNL and secretion of excess hepatic lipids which contributes to the plasma pool of TGs [93].

Fructose consumption as either a late single hit or a double hit did not cause an increase in the levels of cholesterol levels across all treatment groups in both male and female rats. The manifestation of fructose-induced metabolic disorders in adulthood also include hypercholesterolaemia, especially when the high fructose diet is fortified by fats [94]. In the absence of supplemented fat or very high fructose diets, it is uncommon to induce hypercholesterolaemia in rats, a finding which is corroborated by our findings [95, 96].

## Conclusion

We have shown that fructose administration had adverse effects on several health outcomes associated with metabolic dysfunction. The timing (late or double hit) of the administration of fructose had an effect on the development of metabolic dysfunction. We also observed sex-specific differences in the metabolic response to dietary fructose, showing the significance of considering sex effects in metabolic studies. We conclude that neonatal interventional treatment with oleanolic acid during the critical window of developmental plasticity protected against the development of fructose diet-induced health outcomes associated with metabolic dysfunction in male and female Sprague Dawley rats. Following studies in higher animals coupled with molecular analyses to determine its mechanisms, OA should be considered as a natural strategic prophylactic intervention with a lot of potential in the fight against the scourge of metabolic disorders that are impacting significantly on the health systems globally.

## Abbreviations

AESC: Animal Ethics Screening Committee
ANOVA: Analysis of variance
AUC: Area under the curve for the oral glucose tolerance test
CAS: Central animal services
CHOL: Cholesterol
CVDs: Cardiovascular diseases
DMSO: Dimethyl sulphoxide
DNL: De novo lipogenesis
DW: Distilled water
ELISA: Enzyme linked immunosorbent assay
HF: High fructose solution (20% *w*/*v*)
HFCS: High fructose corn syrup
HOMA-IR: Homeostatic model of insulin resistance
IR: Insulin resistance
MetS: Metabolic syndrome
ncRNA: Non-coding ribonucleic acid
OA: Oleanolic acid
OAHF: Oleanolic acid and high fructose solution (20% *w*/*v*) treatment
OGTT: Oral glucose tolerance test
PD: Post-natal day
PGC-1β: Peroxisome proliferator-activated receptor-γ coactivator-1β
ROS: Reactive oxygen species
SRC: Standard rat chow
T2DM: Type 2 diabetes mellitus
TGs: Triglycerides
VAT: Visceral adipose tissue
WHO: World Health Organisation
